# Determination of respiratory gas flow by electrical impedance tomography in an animal model of mechanical ventilation

**DOI:** 10.1186/1471-2466-14-73

**Published:** 2014-04-29

**Authors:** Marc Bodenstein, Stefan Boehme, Stephan Bierschock, Andreas Vogt, Matthias David, Klaus Markstaller

**Affiliations:** 1 Department of Anaesthesiology, University Medical Center Mainz, Mainz 55101, Germany; 2 Department of Anaesthesia, General Hospital of the City of Vienna, Vienna 1090, Austria; 3 Department of Anaesthesiology and Intensive Care Medicine, University Hospital of Cologne, Cologne 50924, Germany; 4 Department of Anaesthesiology and Pain Therapy, Inselspital, Bern University Hospital, and University of Bern, Bern 3010, Switzerland

**Keywords:** Regional respiratory gas flow, Electrical impedance tomography, Mechanical ventilation, Acute respiratory distress syndrome, Acute lung injury, Acute respiratory failure, Intensive care medicine, Spirometry

## Abstract

**Background:**

A recent method determines regional gas flow of the lung by electrical impedance tomography (EIT). The aim of this study is to show the applicability of this method in a porcine model of mechanical ventilation in healthy and diseased lungs. Our primary hypothesis is that global gas flow measured by EIT can be correlated with spirometry. Our secondary hypothesis is that regional analysis of respiratory gas flow delivers physiologically meaningful results.

**Methods:**

In two sets of experiments n = 7 healthy pigs and n = 6 pigs before and after induction of lavage lung injury were investigated. EIT of the lung and spirometry were registered synchronously during ongoing mechanical ventilation. In-vivo aeration of the lung was analysed in four regions-of-interest (ROI) by EIT: 1) global, 2) ventral (non-dependent), 3) middle and 4) dorsal (dependent) ROI. Respiratory gas flow was calculated by the first derivative of the regional aeration curve. Four phases of the respiratory cycle were discriminated. They delivered peak and late inspiratory and expiratory gas flow (PIF, LIF, PEF, LEF) characterizing early or late inspiration or expiration.

**Results:**

Linear regression analysis of EIT and spirometry in healthy pigs revealed a very good correlation measuring peak flow and a good correlation detecting late flow. PIF_EIT_ = 0.702 · PIF_spiro_ + 117.4, r^2^ = 0.809; PEF_EIT_ = 0.690 · PEF_spiro_-124.2, r^2^ = 0.760; LIF_EIT_ = 0.909 · LIF_spiro_ + 27.32, r^2^ = 0.572 and LEF_EIT_ = 0.858 · LEF_spiro_-10.94, r^2^ = 0.647. EIT derived absolute gas flow was generally smaller than data from spirometry. Regional gas flow was distributed heterogeneously during different phases of the respiratory cycle. But, the regional distribution of gas flow stayed stable during different ventilator settings. Moderate lung injury changed the regional pattern of gas flow.

**Conclusions:**

We conclude that the presented method is able to determine global respiratory gas flow of the lung in different phases of the respiratory cycle. Additionally, it delivers meaningful insight into regional pulmonary characteristics, i.e. the regional ability of the lung to take up and to release air.

## Background

Electrical impedance tomography (EIT) can be applied to investigate effect (e.g. recruitment of atelectasis) and side effect (e.g. over distension, cyclical derecruitment) of mechanical ventilation. This technique can be repeated without radiation injury. It has a high temporal resolution and allows at least gross spatial analysis of regional lung mechanics. It can be used as a monitoring technique for regional pulmonary aeration [1]. EIT proved its ability to monitor gas content in any region-of-interest (ROI) in numerous validation studies. It was compared to positron emission tomography [2], single photon emission computed tomography [3], ventilation and perfusion scintigraphy [4], dynamic computed tomography (CT) [5], electron beam CT [6] and spirometry [7].

Up to now EIT was mainly used to quantify regional gas content of the lung during mechanical ventilation or depending on inflation and deflation manoeuvres [8-11]. Quantification of respiratory gas flow by EIT was not described in the present literature. The new approach to observe regional gas flow investigated in this paper uses the high temporal resolution of EIT. Measurement of global or regional gas flow into and out of the lung tissue during different parts of the respiratory cycle might allow description of its mechanical properties depending on lung injury. Specific thresholds that yet have to be determined might define lung disease. Measures of respiratory gas flow might be used together with other measures of EIT to differentiate lung pathology (i.e. bronchial obstruction, atelectasis, hyperinflation). Monitoring of intraindividual changes of regional gas flow during the course of disease might indicate altered functional state of the lung. This information might be used to adapt settings of mechanical ventilation or medication or might lead to further interventions (i.e. bronchoscopy or others).

Respiratory gas flow can be calculated from the course of gas content in a ROI (V_ROI_(t) curve) by its first derivative: V_ROI_’(t). In this study, we describe the regional, dynamic behaviour of the lung (expressed as gas flow) during four different phases of the respiratory cycle:

first phase = early inspiration: peak inspiratory flow (PIF),

second phase = late inspiration: late inspiratory flow (LIF) expressed as the mean flow in this phase,

third phase = early expiration: peak expiratory flow (PEF) and

fourth phase = late expiration: late expiratory flow (LEF) expressed as the mean flow in this phase.

The use of an average value to describe the flow pattern during the late phases of inspiration and expiration represents an approximation in order to allow fast online calculation for potential monitoring purposes in the future.

The aim of this animal experiment was to show applicability of the method to determine regional gas flow in mechanically ventilated pigs. Our primary hypothesis is that global gas flow measured by EIT can be correlated with spirometry. Our secondary hypothesis is that regional analysis of respiratory gas flow delivers meaningful results. The pattern of regional gas flow was described in healthy (applying different ventilator settings) and diseased pigs.

## Methods

With approval of the State Animal Care and Use Committee (Landesuntersuchungsamt Rheinland-Pfalz, 56028 Koblenz, Germany) investigations were done on 13 pigs during general anaesthesia and mechanical ventilation. N = 7 of these 13 pigs were investigated when parameters of ventilation were modified. N = 6 of these 13 pigs were investigated before and after induction of lung injury by lavage.

### Experimental preparation

Experiments were done on 13 pigs weighing 23 ± 3 kg and 12 ± 2 weeks old, split into two experiments. They were sedated by intramuscular injection of 8 mg/kg azaperone (Stresnil®, Janssen-Cilag GmbH, Neuss, Germany), 8 mg/kg ketamine (Ketamin-ratiopharm®, Ratiopharm GmbH, Ulm, Germany) and 0.2 mg/kg midazolam (Midazolam-ratiopharm®, Ratiopharm GmbH, Ulm, Germany). prior to transportation to the animal laboratory. General anesthesia was induced by 4 μg/kg fentanyl (Fentanyl-Janssen®, Janssen-Cilag GmbH, Neuss, Germany), 4 mg/kg propofol (Disoprivan®, Astra Zeneca, London, U.K.) and 0.15 mg/kg pancuronium (Pancuronium-duplex®, DeltaSelect GmbH, Pfullingen, Germany) via peripheral intravenous injection. The pigs were intubated via the orotracheal route (tube diameter: 7.5 mm) and then ventilated with a standard clinical ventilator (Avea®, CareFusion, Höchberg, Germany) in pressure controlled mode with a respiratory rate (RR) of 8 / min, an inspiratory oxygen fraction (FiO_2_) of 0.5 and an inspiration to expiration (I:E) ratio of 1:1. Tidal volume (V_T_) and positive end-expiratory pressure (PEEP) were changed according to the measurement protocol (see below). General anaesthesia was maintained by continuous intravenous administration of 15 mg/kg/h propofol and additional injections of 4 μg/kg fentanyl (e.g. before surgery, before induction of lung injury and before euthanasia). The pigs lay supine throughout the whole experiment. Vascular accesses were achieved by surgical cut-down of the femoral vessels (central venous and arterial line, pulmonary artery catheter).

### Measurement protocol

In the first set of experiments seven healthy pigs were investigated by EIT and spirometry during six ventilation combinations. A V_T_ of 15 or 20 ml/kg reached by a peak inspiratory pressure (PIP) was combined with a PEEP of 0, 5 or 10 mbar. A V_T_ of ≤ 10 ml/kg led to a very low late flow equalling nearly 0 ml/min in pilot experiments.

In the second set of animal experiments six pigs were investigated immediately before and 30 minutes after induction of lung injury. PEEP was set to 5 mbar, V_T_ to 15 ml/kg and FiO_2_ was augmented after induction of saline lavage lung injury if necessary. The injury was induced by surfactant depletion by two consecutive lung lavages with 30 ml/kg warmed, crystalloid solution. After induction of lung injury the pigs were stabilized by fluid and catecholamine (noradrenaline) resuscitation aiming to keep mean arterial pressure above 65 mmHg.

### Electrical impedance tomography (EIT)

A Goe-MF II EIT device (CareFusion, Höchberg, Germany) was used throughout the experiment. Measurements were performed with a temporal resolution of 13 tomograms per second. The thoracic cross section was represented by a matrix of 32 · 32 pixels. Dorsal 8 · 32 pixels of this matrix were not used for evaluation, as they do not contain lung in pig’s anatomy. Ventral 24 · 32 pixels were defined to be the global ROI. The global ROI was further divided into ventral ROI (contains non-dependent lung, first 8 · 32 pixels), middle ROI (second 8 · 32 pixels) and dorsal ROI (contains dependent lung, third 8 · 32 pixels). This method was also described by our group in a recent paper [12].

EIT data were measured and reconstructed by MCFEIT Study Software (Department of Anaesthesiological Research, University of Goettingen, Germany, version 5.02a), and further analysed by AUSPEX (Department of Physics & Medical Technology, VU University Medical Centre, version 1.5).

In all pigs 16 EIT measurement electrodes (Blue sensor®: BR-50-K, Ambu, Bad Nauheim, Germany) were attached around a transversal thoracic layer defining the area to be represented as a tomographic slice. In the cranial caudal axis the height of this measurement layer was defined by an area between sternum and first mamilla. The fold of the axilla was the upper limitation of the ring of electrodes in each pig. In post mortem examinations the expiratory position of the diaphragm was found to be more than 5 cm below the electrode layer. One examination always lasted 60 seconds. Thus 13 · 60 = 780 images were recorded at each time point. Average local impedance of each measurement served as a reference for the reconstruction algorithm (Sheffield backprojection algorithm). Regional, unfiltered, relative impedance change – Z_ROI_(t) – was further analysed in the global, ventral (non-dependent), middle, dorsal (dependent) ROI.

### Calculation of the regional gas flow during four different parts of the respiratory cycle

**Figure 1:**
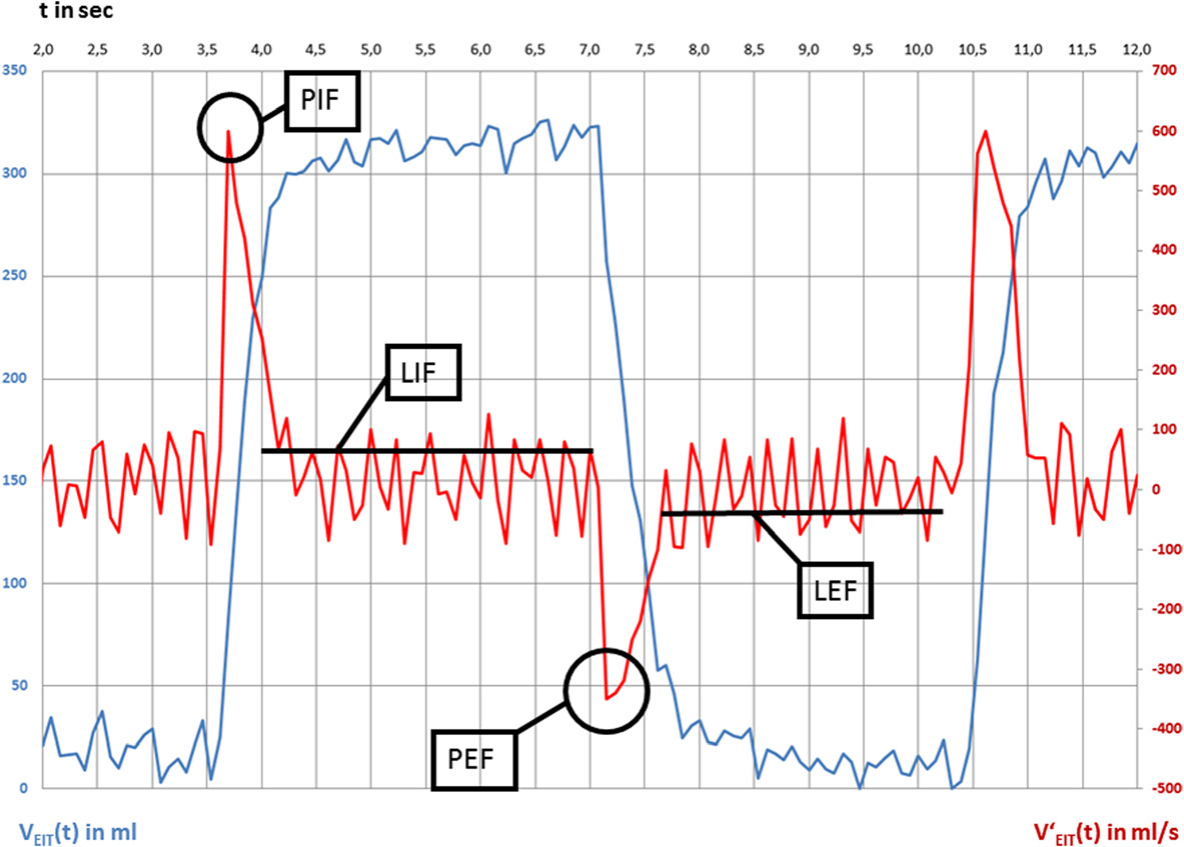
**Determination of gas flow during different parts of the respiratory cycle in an example of EIT data measured during pressure controlled ventilation in the global region-of-interest.** Blue line: measured and calibrated EIT signal, V_EIT_(t) is gas content over time. Red line: calculated flow signal from EIT data, V’_EIT_(t) is gas flow over time. Late inspiratory and expiratory flow (PIF, LIF, PEF and LEF) are derived from gas flow data.

See Figure 1 for illustration. The regional time curves of relative impedance change (Z_ROI_(t), formula 1, dimensionless unit) were calibrated by tidal volume (V_T_) measured by the ventilator and converted to regional volume-time-curves (V_ROI_(t), formula 2, unit in ml).

(1) $$Z_{\mathrm{global}}\left(t\right)=Z_{\mathrm{ventral}}\left(t\right)+Z_{\mathrm{middle}}\left(t\right)+Z_{\mathrm{dorsal}}\left(t\right)$$

(2) $$V_{\mathrm{ROI}}\left(t\right)=Z_{\mathrm{ROI}}\left(t\right)\cdot V_{T}/Z_{T}$$

V_ROI_(t) are V_global_(t), V_ventral_(t), V_middle_(t) und V_dorsal_(t). Z_T_ is the tidal difference (amplitude between inspiration und expiration) of relative impedance changes.

The first derivative of V_ROI_(t) was calculated by standard formula. V_ROI_‘(t) is regional gas flow (formula 3, unit in ml/sec).

(3) $$V_{\mathrm{ROI}}‘\left(t_{n}\right)=\left[V_{\mathrm{ROI}}\left(t_{n}\right)-V_{\mathrm{ROI}}\left(t_{n-1}\right)\right]/\left(t_{n}–t_{n-1}\right)$$

N is any recorded time point, except the first one.

Regional maxima of V_ROI_‘(t) of the respiratory cycle were defined as PIF_ROI_. Peak inspiratory flow was used to describe gas flow in the first phase of the respiratory cycle, i.e. early inspiration. Regional minima of V_ROI_‘(t) were defined as PEF_ROI_. Peak expiratory flow was used to describe gas flow in the third phase of the respiratory cycle, i.e. early expiration.

The whole respiratory cycle lasted 7.5 seconds in our experiment (RR 8/min). Inspiration and expiration each lasted 3.75 seconds (I:E ratio 1:1). The average slope of the V(t) curve was computed during 3.0 seconds starting 0.5 seconds after the time point of PIF_ROI_ or PEF_ROI_. The respective slope of V_ROI_(t) after PIF_ROI_ was defined as LIF_ROI_. Late inspiratory flow was used to describe gas flow in the second phase of the respiratory cycle, i.e. late inspiration. The respective slope of V_ROI_(t) after PEF_ROI_ was defined as LEF_ROI_. Late expiratory flow was used to describe gas flow in the fourth phase of the respiratory cycle, i.e. late expiration. The averaging was used in order to eliminate influences by thoracic blood flow on the signal.

### Spirometry

**Figure 2:**
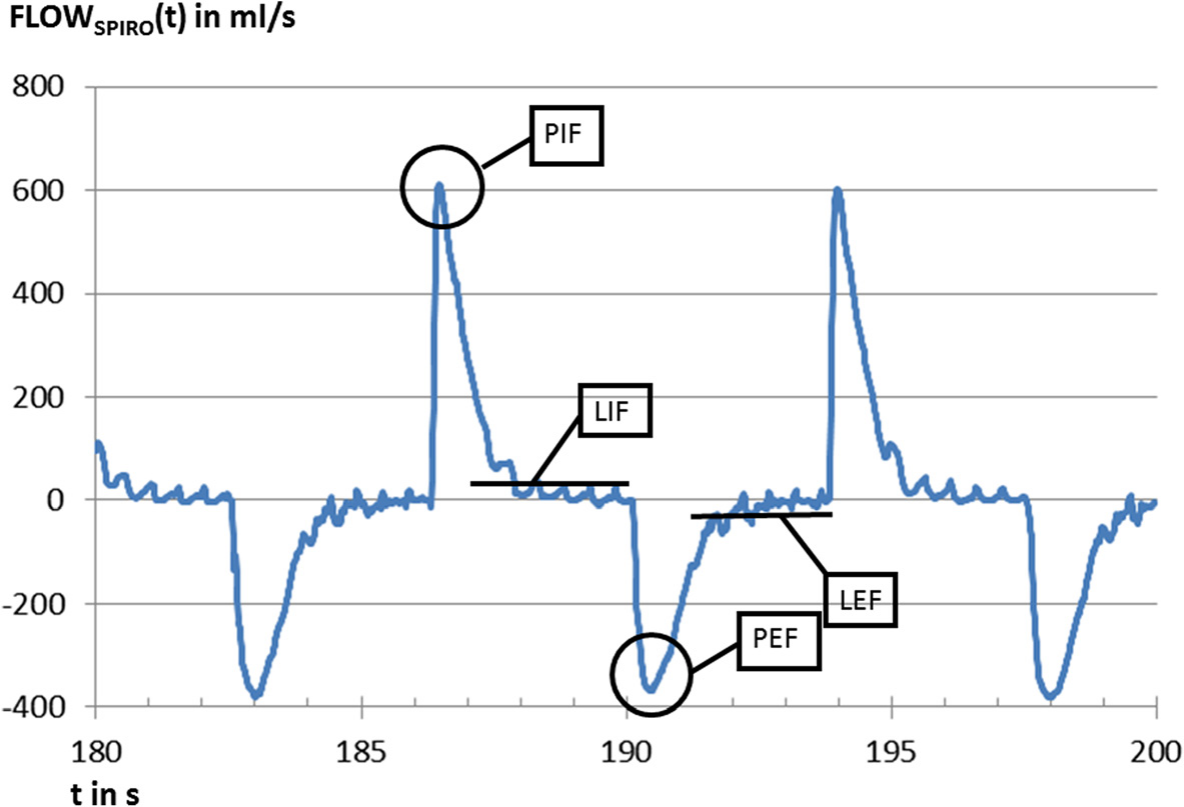
**Example of gas flow measured by spirometry.** FLOWspiro(t). Peak and late inspiratory and expiratory flow (PIF, LIF, PEF and LEF) are used to validate the respective parameters as determined by EIT.

At the beginning of the endotracheal tube a differential pressure flow meter chamber was connected (spirometry module of S/5®, Datex Ohmeda, Duisburg, Germany). Spirometric gas flow was recorded continuously with a frequency of 100 Hz and analysed offline. EIT and spirometry measurements were performed simultaneously. Peak and late inspiratory and expiratory flow (PIF_spiro_, PEF_spiro_, LIF_spiro_ and LEF_spiro_) were selected out of the spirometry data (Figure 2). These results were compared to simultaneously recorded global flow values calculated by EIT (PIF_EIT_, PEF_EIT_, LIF_EIT_ and LEF_EIT_).

### Statistical evaluation

Linear regression analysis was used to correlate gas flow measured by EIT and spirometry. The data was also analysed graphically by Bland-Altman plots. Normal distribution of the results was proved using the test of Shapiro-Wilks. A linear mixed model (two-way ANOVA) was used to link either ventilator setting (first set of experiments) or lung injury (second set of experiments) with the phase-dependent flow in the different ROIs. Multiple testing was adjusted by the Holm–Bonferroni method separated in both experiments. Estimates, 95% confidence intervals and p-values are provided. The multiple tests keep a multiple level of α = 5%. Non-parametric tests for dependent data samples (Wilcoxon test) were used to describe changes of physiological data and of the regional distribution of tidal volume measured by EIT which were induced by lung injury in the second set of experiments. P < 0.05 was considered to be statistically significant. Statistical analysis was performed using SAS 9.2 (SAS Corp. NC, USA).

## Results and discussion

### Validation of gas flow

**Figure 3:**
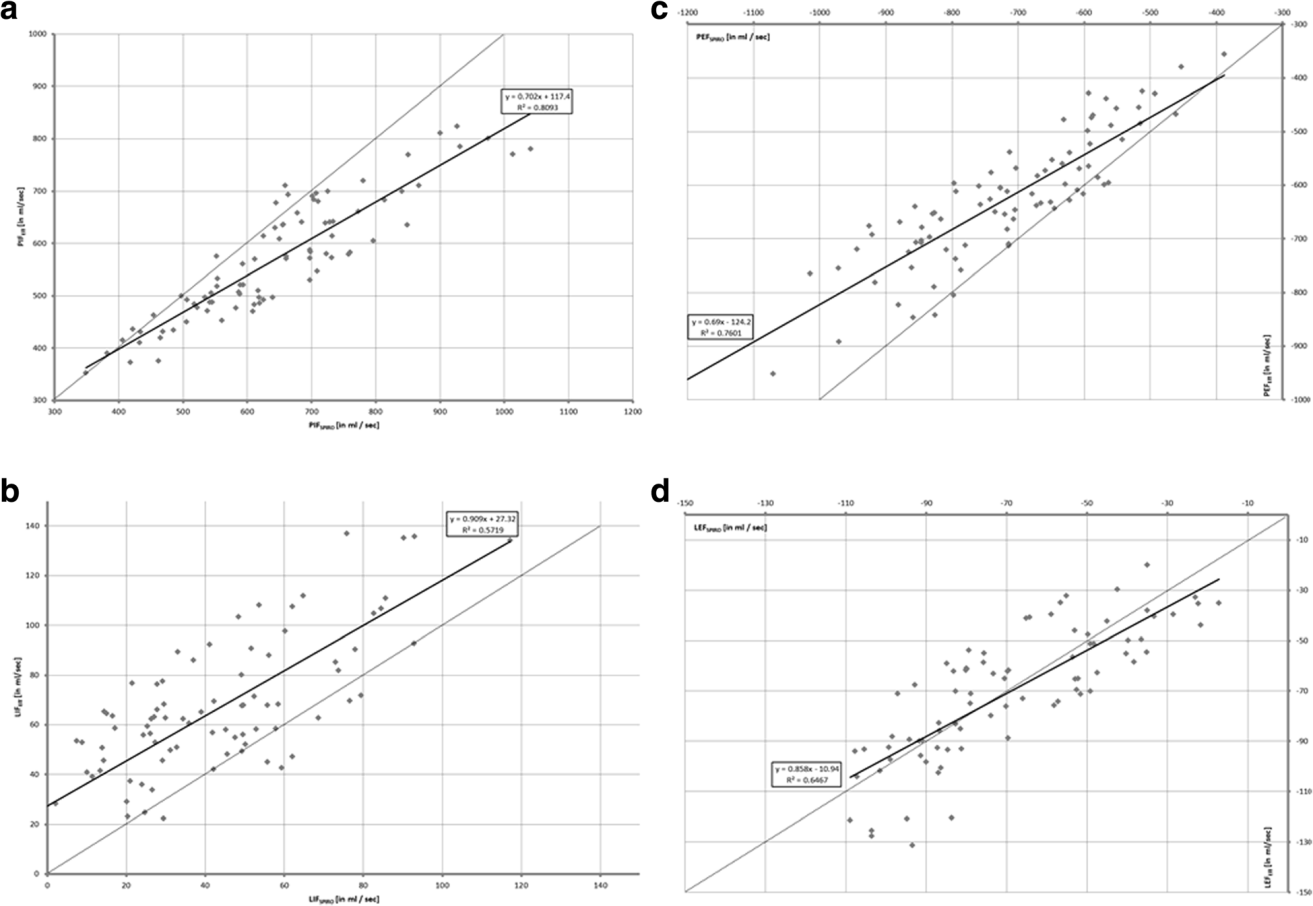
**Linear regression analysis and correlation of gas flow measured by EIT and spirometry.** Dotted line: y = x + 0, black line: linear regression. **a** (left, top). Peak inspiratory flow (PIF). **b** (left, bottom). Late inspiratory flow (LIF). **c** (right, top). Peak expiratory flow (PEF). **d** (right, bottom). Late expiratory flow (LEF).

The linear regression analysis of global gas flow measured by EIT and spirometry depicted the following results (see Figure 3):

1. phase of the respiratory cycle (early inspiration): PIF_EIT_ = 0.702 · PIF_spiro_ + 117.4, r^2^ = 0.809

2. phase of the respiratory cycle (late inspiration): LIF_EIT_ = 0.909 · LIF_spiro_ + 27.32, r^2^ = 0.572

3. phase of the respiratory cycle (early expiration): PEF_EIT_ = 0.690 · PEF_spiro_-124.2, r^2^ = 0.760

4. phase of the respiratory cycle (late expiration): LEF_EIT_ = 0.858 · LEF_spiro_-10.94, r^2^ = 0.647.

**Figure 4:**
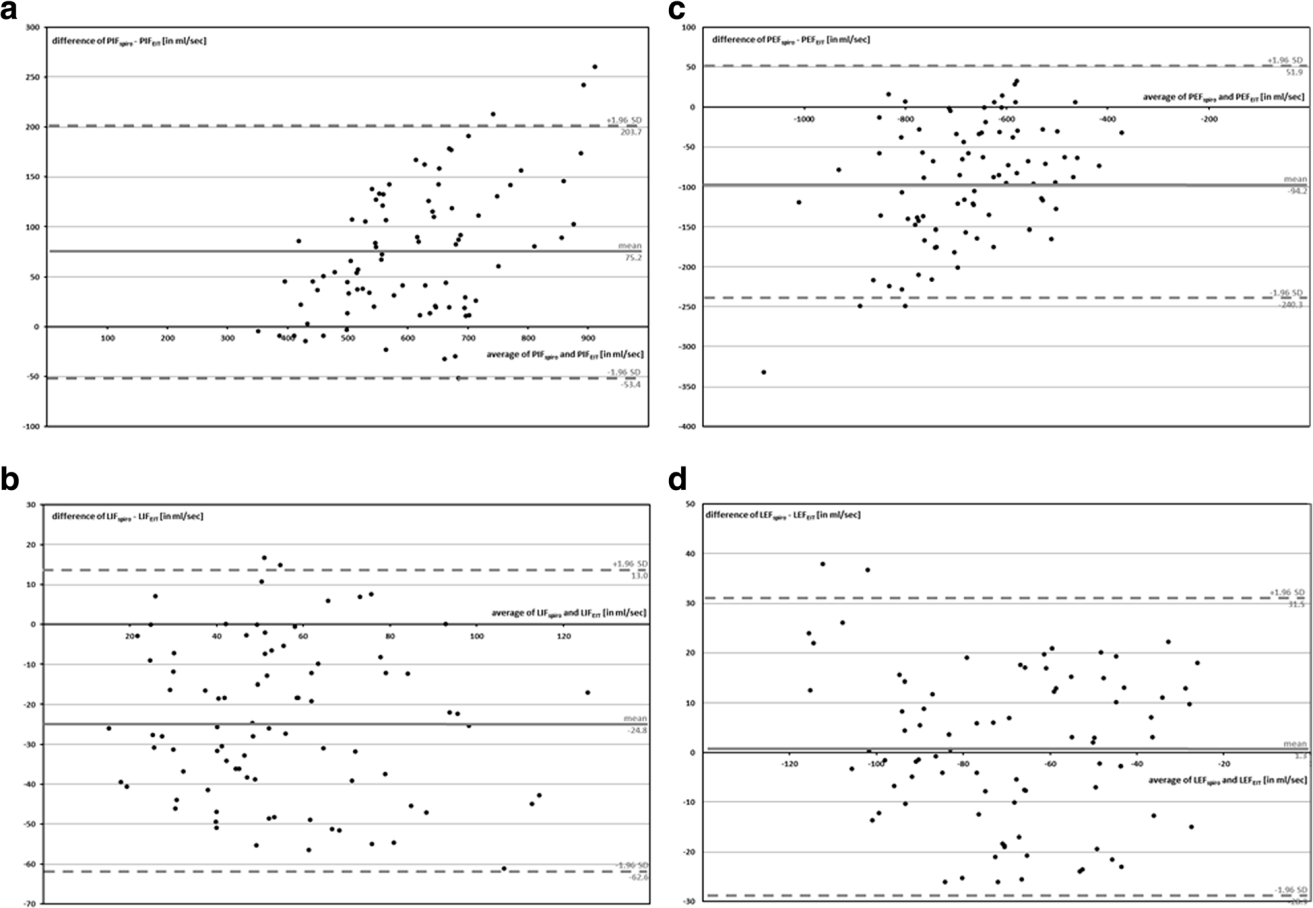
**Bland-Altman plot of gas flow measured by EIT and spirometry. a** (left, top). Peak inspiratory flow (PIF). **b** (left, bottom). Late inspiratory flow (LIF). **c** (right, top). Peak expiratory flow (PEF). **d** (right, bottom). Late expiratory flow (LEF).

Figure 4 contains Bland-Altman plots of global gas flow measured by EIT and spirometry.

Our hypothesis was confirmed. The results of the regression analysis of the global gas flow measured by EIT and spirometry correlated very well or well. But, the correlation of gas flow determined by EIT with spirometry did not reach perfect correlation as it was described in numerous studies for gas content (r^2^ > 0.95) [2-7].

Absolute peak gas flow measured by EIT was generally smaller than measured by spirometry. The Bland-Altman plots reveal a proportional error for PIF and PEF. This can be explained by the different measurement locations of EIT and spirometry and by inertance of the lung tissue. Spirometrical gas flow was measured outside of the body between ventilator and endotracheal tube. EIT depicted the gas flow in the thorax. The EIT signal combines changes of the aeration of the lung tissue and the bronchi. Inert reaction of lung tissue on changes of air content might lead to a reduction of detected peak gas flow by EIT [13].

Late flow was up to 50 times slower than peak flow. Thus, most of the filling and emptying of the lung took place in the first or third phase, represented by peak gas flow. Both, late inspiratory and expiratory gas flow correlated well with spirometry but slightly less reliably than for peak flow measurements.

Two phases of late gas flow separated two phases of peak gas flow during inspiration and expiration. The discrimination of four parts of the respiratory cycle during pressure controlled ventilation proved to be meaningful.

Both methods recorded gas flow using different measurement frequencies (spirometry: 100 measurements per second, EIT: 13 measurements per second). Nevertheless EIT was able to resolve even the fast changes of aeration in early inspiration and expiration. A further increase of accuracy can be expected when higher EIT measurement frequencies are used in modern devices in the future.

### Physiological data

The evaluation of physiological data in both sets of experiments (see Tables 1 and 2) depicts healthy pigs in the first set of experiments (n = 7) and before induction of lung injury in the second set of experiments (n = 6). Heart rate (increase of 41.6%), mean pulmonary artery pressure (increase of 95.0%), oxygenation index (decrease of 67.6%), pulmonary vascular resistance (increase of 111.9%) and dynamic compliance (decrease of 54.0%) changed significantly as a consequence of lung lavage in the second set of experiments. Mean arterial pressure and systemic vascular resistance were not changed significantly, due to sufficient hemodynamic stabilization.

**Table 1:** Physiological data of the animals in the first set of experiments (validation of global gas flow, description of regional gas flow in healthy pigs)

**n = 7**	**Unit**	**Mean**	**sd**
HR	per minute	80.3	10.9
MAP	mmHg	79.3	9.7
MPAP	mmHg	21.7	5.7
PaO_2_ / FiO_2_	mmHg	437.7	89.1
SVR	dyn * s/cm^-5^	1051.6	76.5
PVR	dyn * s/cm^-5^	240.7	53.5
Dyn. Compl.	l/mbar	38.5	11.3

HR = heart rate, MAP = mean arterial pressure, MPAP = mean pulmonary artery pressure, PaO_2_ / FiO_2_ = arterial partial oxygen pressure divided by the fraction of the inspired oxygen, SVR = systemic vascular resistance, PVR = pulmonary vascular resistance, dyn. compl. = dynamic compliance, sd = standard deviation.

**Table 2:** Physiological data of the animals in the second set of experiments (description of regional gas flow in healthy and lavage injured pigs)

	**Healthy baseline**			**Lavage injury**		
**n = 6**	**Unit**	**Mean**	**sd**	**Mean**	**sd**	**p**
HR	per minute	86.2	14.1	122.1	30.1	0.048*
MAP	mmHg	76.1	11.0	71.1	15.6	0.613
MPAP	mmHg	20.1	8.0	39.2	15.1	0.031*
PaO_2_ / FiO_2_	mmHg	444.7	69.7	144.1	45.2	0.002**
SVR	dyn * s/cm^-5^	999.1	111.1	1101.4	114.5	0.333
PVR	dyn * s/cm^-5^	201.1	71.2	426.2	99.1	0.023*
dyn. compl.	l/mbar	38.5	11.3	17.7	12.1	0.039*

HR = heart rate, MAP = mean arterial pressure, MPAP = mean pulmonary artery pressure, PaO_2_ / FiO_2_ = arterial partial oxygen pressure divided by the fraction of the inspired oxygen, SVR = systemic vascular resistance, PVR = pulmonary vascular resistance, dyn. compl. = dynamic compliance, sd = standard deviation, *p < 0.05, **p < 0.005.

### Distribution of tidal volume

The redistribution of tidal volume after induction of lavage injury was subject to evaluation in the second set of experiments (n = 6, see Table 3). The relative part of the tidal volume in the dorsal = dependent ROI decreased significantly after induction of lung injury by lavage (decrease of 68.9%). The relative part of the tidal volume tended to increase in the middle ROI (increase of 31.7%, not statistically significant). The redistribution of tidal volume may reflect pathophysiological changes in the regional lung aeration (i.e. formation of atelectasis).

**Table 3:** Distribution of tidal volume in different regions-of-interest (ROI) in the second set of experiments before and after induction of lung injury measured by EIT

		**Healthy baseline**			**Lavage injury**		
**n = 6**		**Unit**	**Mean**	**sd**	**Mean**	**sd**	**p**
ROI 1	ventral / nondependent	%	15.1	11.1	17.1	16.6	0.647
ROI 2	middle	%	58.3	20.2	76.8	19.5	0.061
ROI 3	dorsal / dependent	%	35.7	13.2	11.1	9.9	0.032*

Values are in% of the total tidal volume (ROI 1 + ROI 2 + ROI 3): sd = standard deviation, *p < 0.05.

### Flow pattern (ROI analysis)

**Figure 5:**
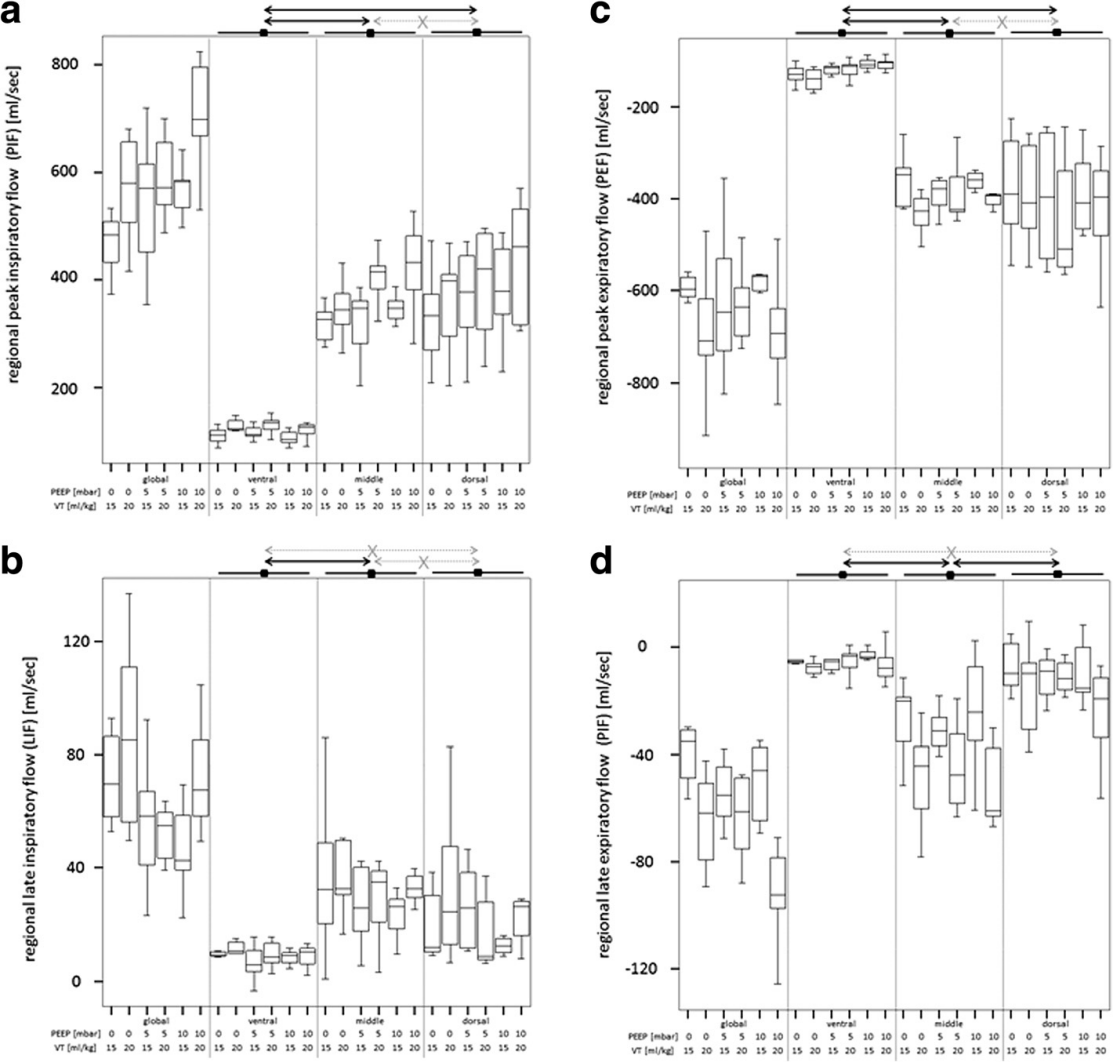
**Pattern of regional gas flow (analysis of global, ventral, middle and dorsal region-of-interest) in n = 7 pigs during pressure controlled ventilation with different ventilator settings.** PEEP = positive end-expiratory pressure. VT = tidal volume. Black arrow: significant group difference. Grey crossed arrow: no significant group difference. **a** (left, top). Peak inspiratory flow (PIF). **b** (left, bottom). Late inspiratory flow (LIF). **c** (right, top). Peak expiratory flow (PEF). **d** (right, bottom). Late expiratory flow (LEF).

**Figure 6:**
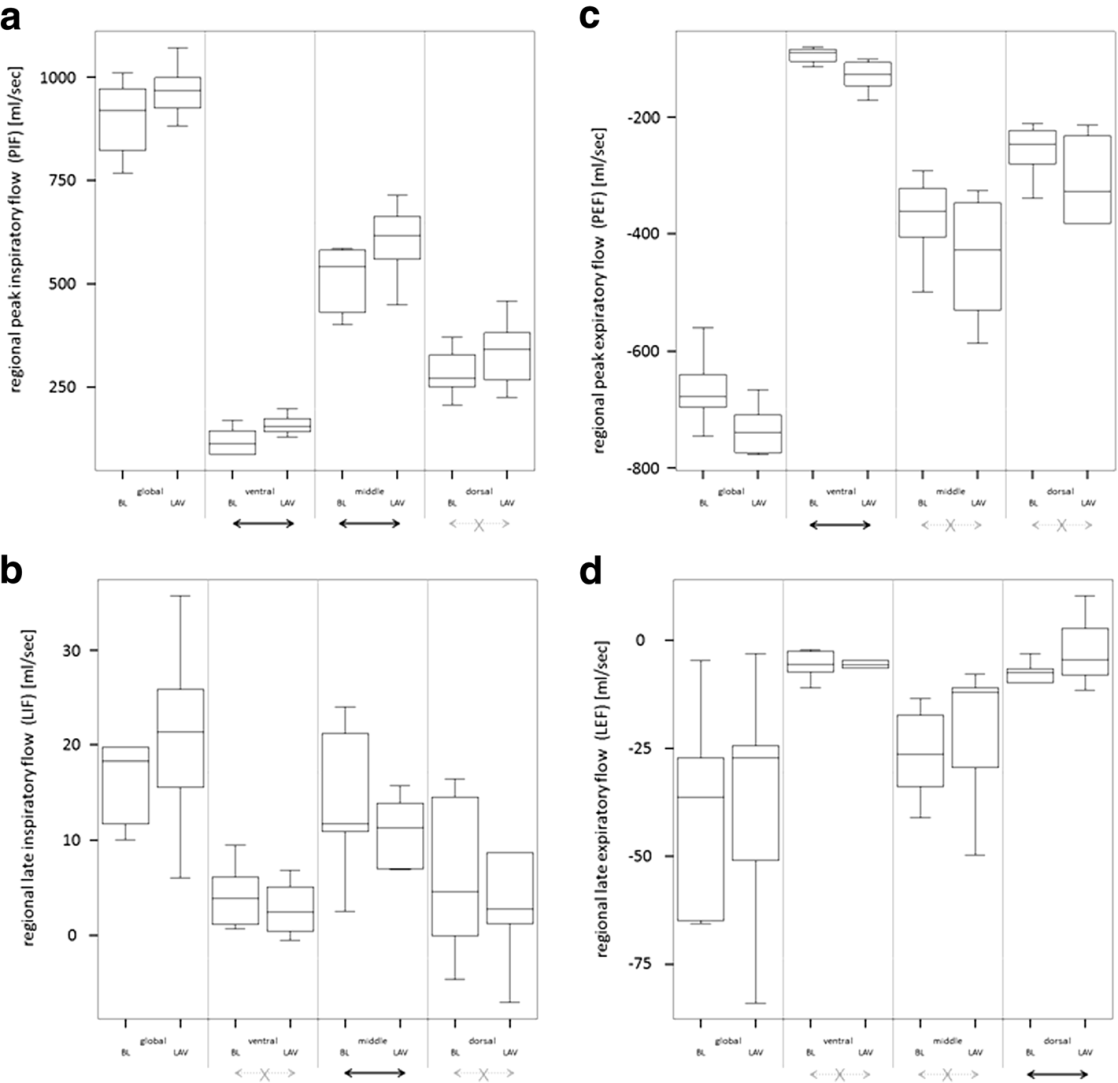
**Pattern of regional gas flow (analysis of global, ventral, middle and dorsal region-of-interest) in n = 6 pigs during pressure controlled ventilation in healthy condition (BL = baseline) and after lung injury induced by lavage (LAV = lavage).** Black arrow: significant difference. Grey crossed arrow: no significant difference. **a** (left, top). Peak inspiratory flow (PIF). **b** (left, bottom). Late inspiratory flow (LIF). **c** (right, top). Peak expiratory flow (PEF). **d** (right, bottom). Late expiratory flow (LEF).

Regional gas flow pattern (PIF_ROI_, LIF_ROI_, PEF_ROI_, LEF_ROI_) in the global, ventral, middle and dorsal ROI from both sets of experiments are listed in Table 4 and are illustrated in Figures 5 and 6. Quantitative results from statistical analysis are listed in Table 5. Global and regional PIF, LIF, PEF and LEF were not influenced by any ventilator settings when compared in the same ROI. But regional PIF, LIF, PEF and LEF were distributed heterogeneously in different ROI, independently from ventilator setting. Ventral ROI generally had a slower PIF and PEF than middle and dorsal ROI. Ventral ROI was generally slower concerning LIF and LEF than the middle ROI. Lavage increased speed of PIF in the ventral and middle ROI and of PEF in the ventral ROI, LIF was reduced in the middle ROI, LEF was slower in the dorsal ROI.

**Table 4:** Peak and late inspiratory and expiratory flow of the lung (PIF, LIF, PEF, LEF) during different parts of the respiratory cycle in different regions-of-interest (ROI)

**PIF**	**Phase 1**	**Inspiration**		**Global ROI**	**Ventral ROI**^**$/§**^	**Middle ROI**	**Dorsal ROI**
**PEEP [mbar]**	**V**_**T**_**/weight [ml/kg]**	**Lung**	**n**	**Mean ± sd [ml/s]**	**Mean ± sd [ml/s]**	**Mean ± sd [ml/s]**	**Mean ± sd [ml/s]**
0	15	healthy	7	467.27 ± 56.69	109.87 ± 15.45	317.60 ± 34.38	298.54 ± 120.62
0	20	healthy	7	571.94 ± 100.47	126.10 ± 17.69	345.79 ± 54.36	327.13 ± 127.57
5	15	healthy	7	510.92 ± 102.16	116.04 ± 14.20	324.25 ± 73.59	334.11 ± 135.32
5	20	healthy	7	621.80 ± 85.20	129.70 ± 16.65	396.75 ± 50.78	348.89 ± 139.71
10	15	healthy	7	550.89 ± 82.35	105.82 ± 13.13	335.46 ± 48.16	339.63 ± 132.18
10	20	healthy	7	711.67 ± 104.68	123.85 ± 22.84	424.07 ± 84.16	402.98 ± 143.06
5	15	healthy	6	901.90 ± 92.06	118.94 ± 32.59	512.73 ± 81.67	283.00 ± 59.44
5	15	LAV	6	968.55 ± 64.49	158.94* ± 24.26	602.78* ± 91.51	335.89 ± 83.67
**LIF**	**Phase 2**	**Inspiration**		**Global ROI**	**Ventral ROI**^**$**^	**Middle ROI**	**Dorsal ROI**
**PEEP [mbar]**	**V**_**T**_**/weight [ml/kg]**	**Lung**	**n**	**Mean ± sd [ml/s]**	**Mean ± sd [ml/s]**	**Mean ± sd [ml/s]**	**Mean ± sd [ml/s]**
0	15	healthy	7	72.04 ± 16.52	10.26 ± 5.27	36.77 ± 28.00	19.03 ± 4.99
0	20	healthy	7	86.66 ± 36.51	10.80 ± 4.08	43.22 ± 24.84	17.66 ± 4.93
5	15	healthy	7	55.58 ± 23.32	5.65 ± 4.35	18.02 ± 3.12	16.05 ± 4.31
5	20	healthy	7	52.11 ± 10.05	9.50 ± 4.76	27.59 ± 15.06	10.45 ± 5.20
10	15	healthy	7	47.17 ± 16.57	7.83 ± 3.67	23.31 ± 8.22	11.93 ± 9.44
10	20	healthy	7	64.37 ± 45.81	8.69 ± 4.00	32.91 ± 5.27	18.07 ± 15.34
5	15	healthy	6	13.58 ± 2.70	4.03 ± 0.52	18.55 ± 8.65	12.72 ± 5.84
5	15	LAV	6	19.09* ± 5.14	2.82 ± 1.83	10.74* ± 5.29	13.02 ± 3.73
**PEF**	**Phase 3**	**Expiration**		**Global ROI**	**Ventral ROI**^**$/§**^	**Middle ROI**	**Dorsal ROI**
**PEEP [mbar]**	**V**_**T **_**/ weight [ml/kg]**	**Lung**	**n**	**Meann ± msd [ml/s]**	**Mean ± sd [ml/s]**	**Mean ± sd [ml/s]**	**Mean ± sd [ml/s]**
0	15	healthy	7	-587.38 ± 86.73	-130.15 ± 21.79	-360.79 ± 60.67	-344.81 ± 140.78
0	20	healthy	7	-687.01 ± 138.84	-141.14 ± 24.29	-419.64 ± 68.85	-360.89 ± 135.22
5	15	healthy	7	-609.64 ± 164.78	-122.24 ± 16.87	-359.02 ± 71.82	-351.49 ± 156.67
5	20	healthy	7	-675.96 ± 142.03	-119.51 ± 20.03	-397.41 ± 67.45	-413.33 ± 170.68
10	15	healthy	7	-581.72 ± 119.23	-107.19 ± 13.15	-356.31 ± 53.46	-353.22 ± 127.59
10	20	healthy	7	-685.25 ± 114.72	-108.40 ± 13.30	-395.27 ± 41.11	-383.60 ± 152.15
5	15	healthy	6	-666.61 ± 62.94	-94.05 ± 12.91	-373.52 ± 74.09	-257.96 ± 47.51
5	15	LAV	6	-734.43* ± 44.74	-130.40* ± 26.63	-441.15 ± 102.11	-349.22 ± 145.95
**LEF**	**Phase 4**	**Expiration**		**Global ROI**	**Ventral ROI**^**$**^	**Middle ROI**^**§**^	**Dorsal ROI**
**PEEP [mbar]**	**V**_**T **_**/ weight [ml/kg]**	**Lung**	**n**	**Mean ± sd [ml/s]**	**Mean ± sd [ml/s]**	**Mean ± sd [ml/s]**	**Mean ± sd [ml/s]**
0	15	healthy	7	-40.23 ± 25.65	-5.74 ± 4.36	-27.25 ± 14.37	-7.11 ± 5.85
0	20	healthy	7	-58.85 ± 30.50	-8.73 ± 4.94	-48.72 ± 19.19	-16.04 ± 7.86
5	15	healthy	7	-50.20 ± 17.22	-5.41 ± 4.37	-27.87 ± 8.07	-3.74 ± 1.78
5	20	healthy	7	-62.46 ± 37.63	-5.46 ± 2.57	-47.50 ± 13.66	-16.79 ± 10.81
10	15	healthy	7	-49.84 ± 37.89	-3.77 ± 2.04	-23.78 ± 22.54	-9.10 ± 11.72
10	20	healthy	7	-86.42 ± 27.98	-6.67 ± 3.74	-51.34 ± 15.72	-15.81 ± 5.00
5	15	healthy	6	-39.16 ± 23.89	-5.71 ± 3.26	-26.44 ± 10.16	-8.36 ± 4.09
5	15	LAV	6	-36.15 ± 27.84	-6.29 ± 2.20	-20.37 ± 16.26	-2.63* ± 1.00

PEEP: positive end-expiratory pressure. V_T_/weight: tidal volume per body weight. sd: standard deviation. LAV: lavage injury. Statistical analysis (two-way ANOVA): *p <0.05: comparison to condition above. ROI analysis: ^$^p < 0.05 comparison to middle ROI, ^§^p < 0.05 comparison to dorsal ROI.

**Table 5:** Results from statistical analysis (two way ANOVA) of the regional flow pattern in both experiments (exp. #1 and #2)

	**ROI/comparison**	**Estimator**	**95% CI**		**P**
**PIF**	ventral < = > middle	-238,8	-275,9	-201,6	**0,008**
exp. #1	ventral < = > dorsal	-223,3	-260,4	-186,2	**0,008**
	middle < = > dorsal	15,4	-79,9	110,8	0,309
**PIF**	ventral: BL < = > LAV	40,0	11,6	68,4	**0,036**
exp. #2	middle: BL < = > LAV	90,1	3,5	176,6	**0,048**
	dorsal: BL < = > LAV	52,9	-18,7	124,4	0,068
**LIF**	ventral < = > middle	-21,5	-30,7	-12,3	**0,021**
exp. #1	ventral < = > dorsal	-6,7	-16,0	2,5	0,068
	middle < = > dorsal	14,8	-4,0	25,5	0,073
**LIF**	ventral: BL < = > LAV	-1,2	-2,4	0,0	0,097
exp. #2	middle: BL < = > LAV	-7,8	-14,8	-0,8	**0,045**
	dorsal: BL < = > LAV	0,3	-4,5	5,1	0,798
**PEF**	ventral < = > middle	260,0	220,6	299,4	**0,008**
exp. #1	ventral < = > dorsal	246,5	207,1	285,9	**0,008**
	middle < = > dorsal	-13,5	-117,4	90,4	0,384
**PEF**	ventral: BL < = > LAV	-36,4	-56,1	-16,6	**0,027**
exp. #2	middle: BL < = > LAV	-67,6	-155,7	20,5	0,065
	dorsal: BL < = > LAV	-91,3	-188,0	5,5	0,053
**LEF**	ventral < = > middle	31,8	22,1	41,4	**0,015**
exp. #1	ventral < = > dorsal	5,5	-4,2	15,1	0,088
	middle < = > dorsal	-26,3	-37,7	-14,9	**0,022**
**LEF**	ventral: BL < = > LAV	-0,6	-3,3	2,2	0,235
exp. #2	middle: BL < = > LAV	6,1	-7,1	19,3	0,109
	dorsal: BL < = > LAV	5,7	3,2	8,3	**0,022**

Peak and late inspiratory and expiratory flow of the lung (PIF, LIF, PEF, LEF) in the ventral, middle and dorsal region-of-interest (ROI). BL: healthy baseline, LAV: lavage injury, CI: confidence interval, P: bold printed test results reflect statistically significant differences (P<0.05).

Physiological considerations allow interpretation of the ROI analysis. Non-dependent regions received a slower peak flow than dependent areas, hyperinflation in the ventral area might partially explain this effect [14]. Flow was generally very slow (often close to 0) in the ventral ROI and in the dorsal ROI during the late phases of the respiratory cycle. This finding indicates complete filling or emptying during early inspiration or expiration in the non-dependent and dependent ROI. Detectable late flow in the middle ROI indicated incomplete filling or emptying during early inspiration or expiration. But, respiratory flow was much higher in the early phases than in the late phases. Respiratory mechanics of the middle ROI allowed gas flow throughout the respiratory cycle indicating best regional mechanical characteristics.

Investigated ventilator settings (changes of PEEP and VT) had neither relevant nor significant influence on gas flow pattern in healthy lungs.

Lung lavage led to changes in gas flow pattern. These changes were moderate in size. Probably early, still recruitable atelectasis was the dominating pathology. Ventral and middle ROI had better mechanical properties after lung injury than the dorsal ROI: peak inspiratory gas flow increased here after lung lavage in inspiration. In expiration higher lung weight might have accelerated peak emptying after opening the expiratory valves. Again ventral ROI was faster than before lung injury. Dorsal ROI was hit in late expiration, as expected, probably due to atelectasis [14,15].

The presented data have some limitations.

The influence of the I:E ratio and the respiratory rate on gas flow needs to be addressed, as time dependent changes were not made in the present study.

Representative and summarizing flow values were chosen to describe the early phases (peak flow) and the late phases of inspiration and expiration (mean flow). This approach has the advantage to be easily computable, especially if this method is used for an online pixel-wise representation of regional flow in the future. But this approach represents only a descriptive approximation of the real, non-linear flow pattern during these four phases.

As stated above only the global peak and mean flow of early and late phases of the inspiration and expiration measured by EIT were validated by our data. Nevertheless regional flow measured by EIT and calculated by our method was presented in this manuscript. Presently, there is no other method to compare these EIT derived regional flow values to. Measurement of regional gas content by EIT is valid [2-7]. Additionally, the time resolution of its measurement is rather high allowing calculation of its first derivative. Thus, the principle of the chosen method is estimated to deliver reliable results.

The content of lung tissue may differ between the investigated ROIs. Although the ROIs have equal sizes they might contain different amounts of lung tissue. Especially the amount of lung tissue in the ventral ROI might differ from the other ROIs [16,17]. The ROI analysis did not exclude the central (mediastinum, heart) region of the lung. This area does not take up or release gas. Thus, its contribution to our findings is low concerning flow. Changes of size, blood content and location of the central thoracic region between inspiration and expiration are low. Thus, their influence on electrical characteristics of the EIT measurement during inspiration and expiration is negligible [1].

We investigated our method in an animal experiment applying tidal volumes of 15 or 20 ml/kg bodyweight. This was necessary to measure late flow (see above). Thus our method is not validated in a clinically relevant range of tidal volumes around 6 ml/kg.

The presented method allows a new insight into regional lung mechanics. It may be included in automatic, online EIT evaluation tools. Color-coded tomograms of pixel-wise calculated gas flow from the different phases of the respiratory cycle would allow a detailed ROI analysis. More data is needed to understand the meaning of regional flow pattern, especially the nature of regional pulmonary inertance. The next step can be a comparison of different lung injury models at different time points in order to use these values to describe specific lung pathology. A future investigation of regional gas flow during absence of gravity may discriminate between anatomical and physiological reasons for inertance [18,19].

## Conclusions

A new method to calculate regional respiratory gas flow using the first derivative of the regional aeration signal measured by EIT was introduced and validated on a global level. The discrimination of four phases of the respiratory cycle proved to be meaningful. Regional analysis of the flow pattern depicted heterogeneous velocities of gas filling or emptying. This pattern was influenced by early, moderate lavage injury. Non-invasive determination of gas flow proved to be possible during different phases of the respiratory cycle by EIT during ongoing mechanical ventilation.

## Abbreviations

ARDS: Acute respiratory distress syndrome; CT: Computed tomography; EIT: Electrical impedance tomography; FiO2: Inspiratory fraction of oxygen; I:E ratio: Inspiratory to expiratory ratio; LEF: Late expiratory flow; LIF: Late inspiratory flow; PEEP: Positive end-expiratory pressure; PEF: Peak expiratory flow; PIF: Peak inspiratory flow; ROI: Region of interest; RR: Respiratory rate; VT: Tidal volume; VROI(t): Regional gas content; VROI’(t): Regional gas flow (first derivative of V_ROI_(t)).

## Competing interests

The authors declare that they have no competing interests.

## Authors’ contributions

MB is the principal investigator and is the corresponding author. He carried out the experimental design and performed the measurements, the data evaluation and statistical analysis, he drafted the manuscript. SBo participated during the experiments and the data evaluation, he helped to draft the manuscript. SBi participated during the experiments and the data evaluation. He helped to draft the manuscript. AV participated during the experiments and helped to draft the manuscript. MD was involved in the experimental design and he helped to draft the manuscpript. KM initiated the study and was involved in the experimental design. He supervised the experiments and the data evaluation and corrected the manuscript. All authors read and approved the final manuscript.

## Pre-publication history

The pre-publication history for this paper can be accessed here:

http://www.biomedcentral.com/1471-2466/14/73/prepub
